# Induction of p53-inducible microRNA miR-34 by gamma radiation and bleomycin are different

**DOI:** 10.3389/fgene.2012.00220

**Published:** 2012-10-19

**Authors:** Ufuk Mert, Emre Özgür, Duygu Tiryakioglu, Nejay Dalay, Ugur Gezer

**Affiliations:** Department of Basic Oncology, Oncology Institute, Istanbul UniversityIstanbul, Turkey

**Keywords:** genotoxic stress, bleomycin, γ-radiation, p53, miR-34, gene expression

## Abstract

microRNAs (miRNAs) are small molecules in their mature form and master regulators of gene expression. Recent work has shown that miRNAs are involved in the p53 network. Of the various miRNAs, miR-34 is regulated by the p53 protein. miR-34 can be induced by ionizing radiation (IR) *in vitro* and *in vivo*. However, there is no data in the literature for induction of miR-34 by a chemical agent inducing DNA damage. Here we studied the expression of miR-34 in HeLa and MCF-7 cells exposed to genotoxic stress-induced by bleomycin (BLM) or γ-radiation. We first analyzed p53 accumulation upon DNA damage induction. The basal level of p53 in MCF-7 cells was higher (approx. 6-fold) than in HeLa cells, and its accumulation was similar for both DNA-damaging agents in both cell lines. We have shown that miR-34 is significantly induced by γ-radiation in HeLa cells, but not in MCF-7 cells. BLM did not significantly affect miR-34 expression in both cell types. In conclusion, our findings reveal that miR-34 induction by genotoxic stress may be cell-type specific.

## Introduction

Eucaryotic genomes encode thousands of non-coding RNA molecules (ncRNAs). microRNAs (miRNAs) constitute a major type of the untranslated RNA molecules. These are small molecules in their mature form (<25 nt) and master regulators of gene expression. By degrading or blocking translation of target messenger RNA molecules, these small miRNAs can regulate expression of more than half of all protein-coding genes in the mammalian genomes (Cortez et al., 2010). Aberrant miRNA expression is well-characterized in cancer initiation/progression and may also have prognostic implications in cancer (Lovat et al., 2011).

Recent work has shown that miRNAs are important components in the p53 network (Feng et al., 2011). Of the various miRNAs, miR-34 has been found to be regulated by the p53 protein (Chang et al., 2007; He et al., 2007; Tarasov et al., 2007). Expression of miR-34 is greatly induced by DNA damage and oncogenic stress in a p53-dependent manner (He et al., 2007). This tumor suppressor miRNA exerts potent anti-proliferative effects, as an increase in its expression may induce cell-cycle arrest, apoptosis or senescence (Hermeking, 2010). miR-34 is inactivated by aberrant CpG methylation in multiple tumor types (Lodygin et al., 2008). However, recent data suggest that alteration of miR-34 expression may also occur, at least partially, independent of p53 regulation (Chen et al., 2011).

miR-34 can be induced by ionizing radiation (IR) *in vitro* (He et al., 2007) and *in vivo* (Liu et al., 2011). However, there is no data for induction of miR-34 by a chemical agent which induces a similar DNA damage pattern as IR. Therefore, we studied the expression of miR-34 in HeLa and MCF-7 cells exposed to genotoxic stress-induced by bleomycin (BLM) and γ-radiation and correlated it with p53 accumulation.

## Materials and methods

### Cell culture and DNA damage induction

The study was conducted using HeLa and MCF-7 cells which were purchased from the German Resource Centre for Biological Materials (DSMZ). Cells were grown in the DMEM culture medium (Biochrom, Berlin, Germany) supplemented with 10% FCS under standard conditions. Experiments were performed with cells with a passage number <20. For DNA damage induction, 4 × 10^5^ cells were plated into 60 mm petri dishes. Twenty-four hours after plating BLM (Applichem, Darmstadt, Germany) was added at doses of 0, 37.5, and 75 μg/ml or the cells were irradiated to the total doses of 0, 2, and 5 Gy using a Cobalt-60 γ-ray source at a dose rate of 125 cGy/min and kept under standard growth conditions for further 24 h.

### Measurement of the p53 protein levels

We measured the p53 protein levels upon exposure of the cells to genotoxic agents by a pan-p53 ELISA assay (Roche, Mannheim, Germany) according to the manufacturer's instructions. The assay was performed using cytoplasmic lysates, and relative p53 concentrations were determined from the mean absorbance values after constructing a calibration plot.

### miR-34 expression analysis

Using a commercial kit (Qiagen, Valencia, CA, USA) we simultaneously extracted small and long RNAs from cells. This was achieved by selective elution steps of RNA molecules from silica membranes. Small RNA molecules were converted to cDNA using a specific kit (Qiagen) including poly-A polymerase and reverse transcriptase enzymes. miR-195 was co-amplified as a control molecule for which no association to DNA damage or p53 has been shown. Results were standardized to miR-10b. miRNAs were quantified using SYBR Green (Roche) as the fluorescent dye. qPCR was performed in the LightCycler 480 Instrument (Roche), and samples with a Ct >40 were considered negative. Amplification of the appropriate product was confirmed by melting curve analysis following the amplification reaction.

### Statistics

Statistical analysis was performed using Student's *t*-test, and *p* < 0.05 was considered statistically significant. Three-independent cell culture experiments were used to calculate the average value of the p53 or miR-34 expression.

## Results

**Figure 1:**
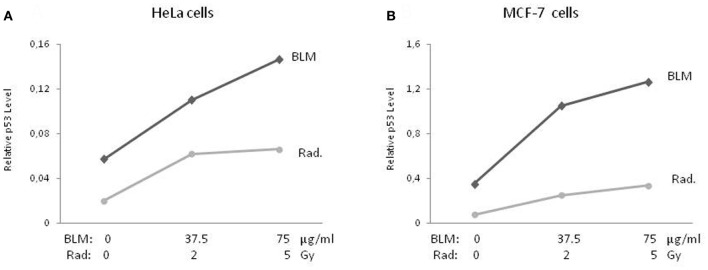
**Accumulation of p53 protein.** Cytoplasmic lysates of BLM-treated and irradiated cells were used to quantitatively determine the accumulation of p53 protein (**A** and **B**) by ELISA. Results of three-independent cell culture experiments were evaluated to calculate the average value. BLM, bleomycin; Rad., radiation.

p53 protein levels were measured 24 h after adding BLM to the medium or irradiation of the cells. The basal p53 expression level in the MCF-7 cells was higher (approx. 6-fold) than the in HeLa cells. As seen in Figure 1, a similar p53 accumulation pattern was observed in response to both DNA-damaging treatments in both cell lines. BLM treatment with doses of 37.5 and 75 μg/mL led to an 1.92 and 2.56-fold increase in the amount of p53 in HeLa cells, respectively, when compared to untreated cells (Figure 1A). For irradiation, the rates were 3 and 3.3-fold for doses of 2 and 5 Gy, respectively. In the MCF-7 cells (Figure 1B), the increase in the p53 levels was slightly higher (2.98 and 3.6-fold) for the indicated BLM doses when compared to radiation (3.28 and 4.46-fold).

**Figure 2:**
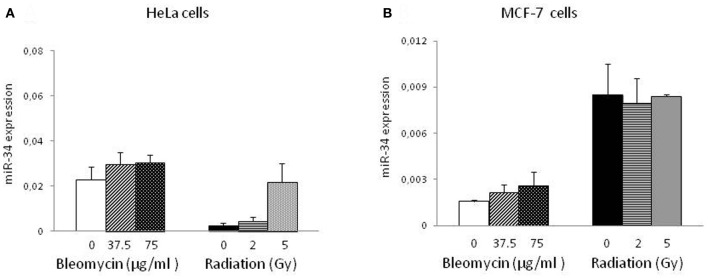
**miR-34 expression in HeLa and MCF-7 cells.** miR-34 levels were measured by quantitative PCR in HeLa **(A)** and MCF-7 **(B)** cells exposed to genotoxic stress. Results of three-independent cell culture experiments were evaluated to calculate the average value of relative gene expression. Each column represents mean + S.E.

Subsequently, we investigated the expression of miR-34 at the same time point as above. Interestingly, miR-34 was significantly induced only by radiation in HeLa cells (Figure 2A) but not in MCF-7 cells (Figure 2B). BLM-induced DNA damage did not result in an increase of miR-34 levels in both cell lines. In irradiated HeLa cells miR-34 levels were elevated approx. 2 and 14-fold following radiation at doses of 2 and 5 Gy, respectively (*p* < 0.01). In contrast to miR-34, levels of miR-195 were not affected by genotoxic stress (data not shown) indicating the specificity of the induction.

## Discussion

Recent work has revealed that miRNAs are involved in the p53 network (Feng et al., 2011). miR-34 is regulated by the p53 protein and induces apoptosis and G1-arrest (Tarasov et al., 2007). This molecule can be induced by IR *in vitro* (He et al., 2007) and *in vivo* (Liu et al., 2011). However, there is no data for the induction of miR-34 by a DNA damaging chemical agent. In this study our aim was to assess the expression of miR-34 in HeLa and MCF-7 cells when exposed to genotoxic stress-induced by BLM or γ-radiation. When the two cell lines were analyzed, it was noteworthy that HeLa cells had lower basal levels of p53. This may be associated with human papilloma virus (HPV) infection and consequent degradation of p53 via the E6 protein in these cells (Hoppe-Seyler and Butz, 1993). Despite the lower levels, genotoxic stress led to a similar accumulation pattern of the p53 protein HeLa cells as in MCF-7 cells.

Although p53 accumulated upon DNA damage by both agents in both cell lines, induction of miR-34 by BLM and IR was different. It was significantly induced by radiation-induced DNA damage only in HeLa cells but not in MCF-7 cells. BLM treatment did not affect the miR-34 expression. These findings suggest that the miR-34 molecule is regulated by p53 in a cell-type specific manner. As MCF-7 cells are known to be resistant to radiation-induced intrinsic apoptotic cell death (Jänicke et al., 2001; Özgür et al., 2012), it is plausible to assume that miR-34 may not be essential in these cells. On the other hand, the failure of BLM to induce miR-34 may be explained by the fact that the p53 pathway functions differentially in stress conditions caused by various agents and that it can induce cell cycle arrest or apoptosis through alternative mechanisms. We have recently shown that BLM and γ-radiation lead to a differential expression of some long non-coding RNAs (Özgür et al., 2012). On the other hand, it was shown that miR-34 expression is at least partially independent of p53 regulation (Chen et al., 2011). These data suggest that miR-34 may be regulated in a complex way by different mechanisms which should be elucidated in future studies.

### Conflict of interest statement

The authors declare that the research was conducted in the absence of any commercial or financial relationships that could be construed as a potential conflict of interest.
